# Biocompatible Magnetic Conjugated Polymer Nanoparticles for Optical and Lifetime Imaging Applications in the First Biological Window

**DOI:** 10.1021/acsapm.2c01153

**Published:** 2022-10-12

**Authors:** Struan Bourke, Federico Donà, Yurema Teijeiro Gonzalez, Basma Qazi Chaudhry, Maryna Panamarova, Eirinn Mackay, Peter S. Zammit, Lea Ann Dailey, Ulrike S. Eggert, Klaus Suhling, Mark A. Green

**Affiliations:** † Department of Physics, King′s College London, London WC2R 2LS, U.K.; ‡ Randall Centre for Cell and Molecular Biophysics, Faculty of Life Sciences and Medicine, King’s College London, London SE1 1UL, U.K.; § Department of Cell and Developmental Biology, University College London, Gower Street, London WC1E 6BT, U.K.; ∥ Department of Pharmaceutical Technology and Biopharmaceutics, University of Vienna, Universitätsring 1, 1010 Vienna, Austria

**Keywords:** conjugated polymers, nanoparticles, biological imaging, cytotoxicity studies, fluorescence lifetime studies, zebrafish

## Abstract

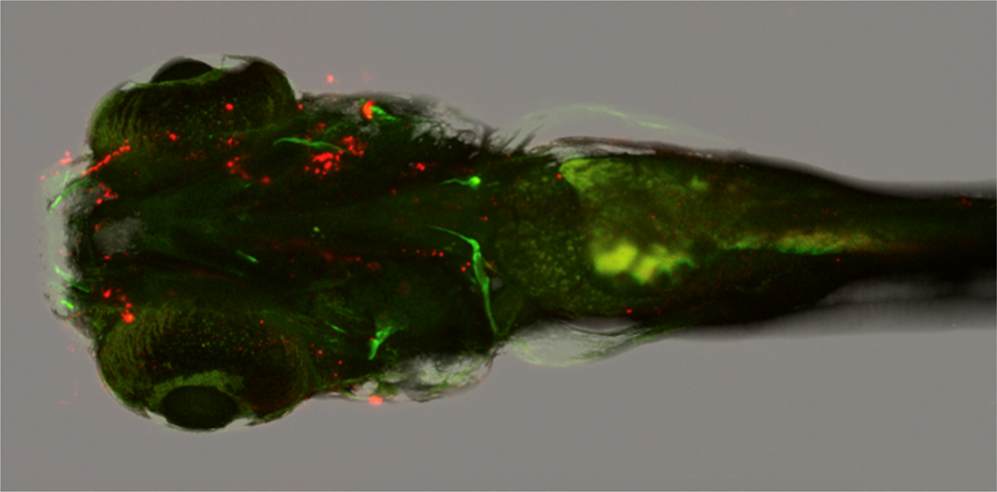

Conjugated polymers are organic semiconductors that can be used for fluorescence microscopy of living specimens. Here, we report the encapsulation of the bright-red-emitting conjugated polymer, poly[{9,9-dihexyl-2,7-bis(1-cyanovinylene)fluorenylene}-alt-co-{2,5-bis(*N*,*N*′-diphenylamino)-1,4-phenylene}] (CN-FO-DPD), and superparamagnetic iron oxide nanoparticles (SPIONs) within poly(styrene-*co*-maleic anhydride) (PSMA) micelles. The resulting particles exhibited an emission peak at 657 nm, a fluorescence quantum yield of 21%, an average diameter of 65 nm, and a ζ potential of −30 mV. They are taken up by cells, and we describe their use in fluorescence microscopy of living Hela cells and zebrafish embryos and their associated cytotoxicity in HEK, HeLa, and HCE cells.

## Introduction

In recent years, medical imaging has seen improvements in a wide array of available techniques, instrumentation, as well as contrast agents used. This has ranged from better image resolution and more sensitive detectors to enhanced identification of pathological markers that leads to improved treatments. There are a number of different means to achieve this, and the field of nanotechnology has provided tools for drug delivery, biosensors, and labeling agents.^1,2^ Current fluorescence microscopy and medical imaging often employ organic fluorescent dyes, which are limited in both their brightness and rapid photobleaching.^3^ Alternatively, quantum dots (QDs) overcome a number of these issues but are usually composed of heavy metals that limits their use in clinical imaging.^4−6^ Therefore, materials need to be brightly fluorescent, stable, and biocompatible to be used for fluorescence microscopy and imaging.^7^ For use in biological imaging, materials with optical properties associated with red/near-infrared spectral regions are of immense interest, as skin and blood are relatively transparent in these ranges, notably between *ca.* 650 and 1350 nm, termed the first near-infrared window. The development of stable, simple, emissive material with optical properties in this spectral region is a key goal for imaging scientists and clinicians.

Conjugated polymers, organic semiconductors capable of light harvesting and emission, have been shown to be effective in optoelectronics,^8,9^ photovoltaics,^10,11^ sensing, and imaging.^12−14^ However, due to being inherently hydrophobic in nature, they need to be prepared as nanoparticles, also referred to as polymer dots (P-dots) and semiconductor polymer nanoparticles (SPNs).^12,15−19^ There are a number of advantages when using conjugated polymer nanoparticles (CPNs) over QDs, including their ease of processing, large extinction coefficients, and biologically inert components circumventing the issue of heavy-metal toxicity in QDs.^20,21^ CPNs have great potential as biological imaging tools and can be further engineered to act as drug delivery systems, carrying anticancer drugs such as doxorubicin^22^ or camptothecin.^23^ There are reports of CPNs doped with near-infrared (NIR) dyes^24^ and photodynamic therapy (PDT) agents such as meta-tetra(hydroxyphenyl)-chlorin (m-THPC),^25^ chlorin e6,^26^ and porphyrins.^27^ There are also a number of reports^28,29^ on modified conjugated polymers that can emit in the NIR/IR region that removes the need for dopants, whereas other similar reports describe utilizing conjugated polymers as photosensitizers.^30,31^ CPNs can also incorporate other nonemissive dopants such as iron oxide nanoparticles for multimodal magnetic resonance imaging.^32−34^ The addition of iron oxide allows the CPNs to respond to an external magnetic field, which can assist in the purification of nanoparticles during synthesis, avoiding the use of time-consuming dialysis or expensive filtration.

Here, we report the use of poly[{9,9-dihexyl-2,7-bis(1-cyanovinylene)fluorenylene}-alt-co-{2,5-bis(*N*,*N*′-diphenylamino)-1,4-phenylene}] (CN-FO-DPD), a commercially available conjugated polymer with emission just inside the first biological window (*ca*. 650–950 nm), combined with iron oxide in the synthesis of brightly emitting biocompatible nanoparticles. We report their optical and physical characteristics and biocompatible nature, as well as describe their use in cellular imaging.

## Results and Discussion

**Figure 1:**
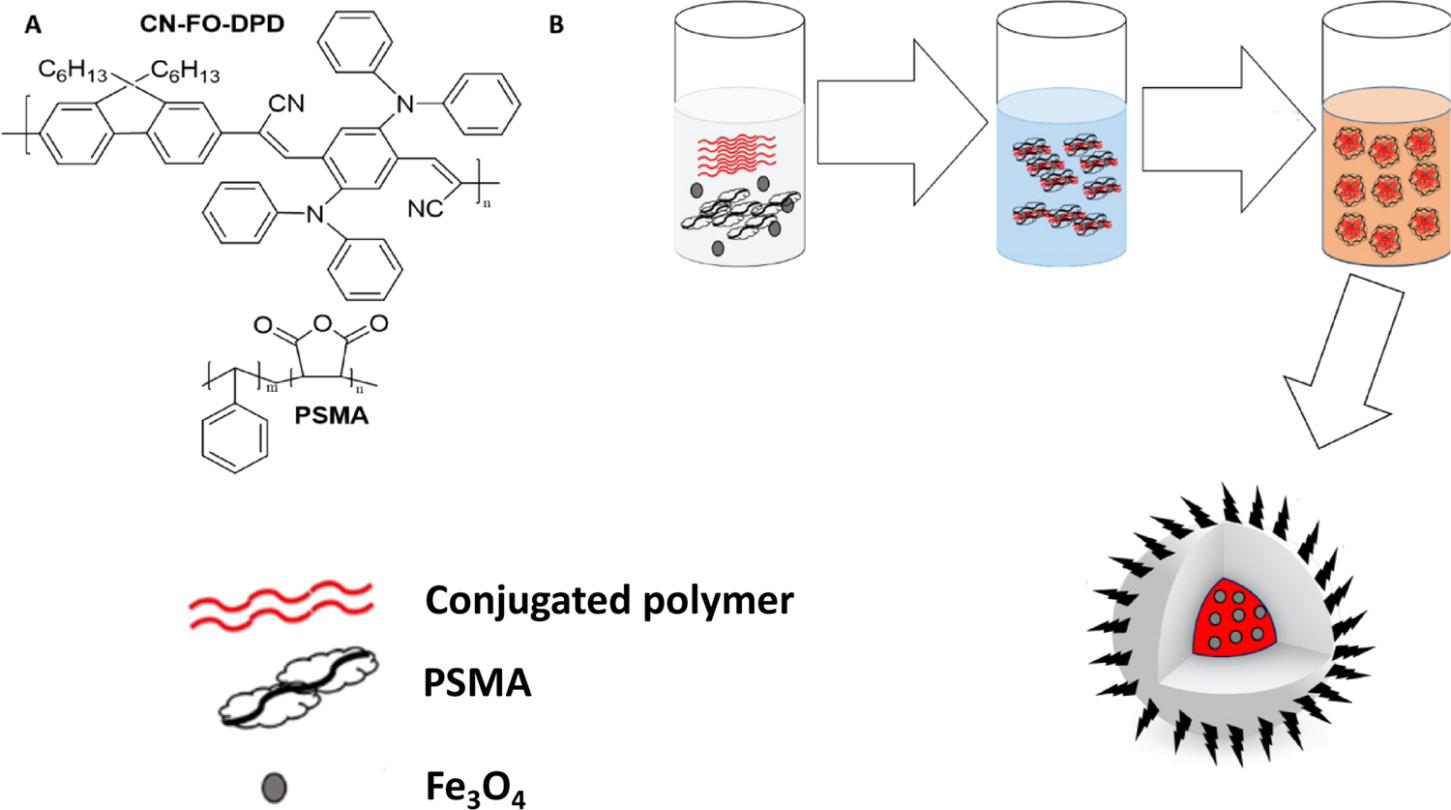
(A) Chemical structures of CN-FO-DPD and PSMA. (B) Schematic illustration of the preparation of the CN-FO-DPD/iron oxide nanoparticles encapsulated in PSMA.

The chemical structure of CN-FO-DPD is presented in Figure 1A. The magnetic, red-emitting CPNs were prepared by the modified reprecipitation method (Figure 1B).^35^ The resulting nanoparticle dispersion was bright red and appeared stable and clear for at least a month without aggregation (Figure S1). By including magnetic iron oxide particles concurrently with the conjugated polymer and PSMA during preparation, SPIONs were encapsulated inside PSMA, chosen due to its amphiphilic nature and its ability to encapsulate the hydrophobic species while presenting carboxylic acid group on the particle’s exterior. In previous cases of CPN preparation, the optical properties of the conjugated polymers were found to be dependent on the initial concentration of polymer, the solvent, and the physical conformation of the chains.^36^ In the case of this conjugated polymer, the optical properties were unaffected by the starting concentration and an effective, stable red emitter was realized due to the constant particle size obtained with varying reaction conditions.

### Optical Characterization of Conjugated Polymer Nanoparticles

**Figure 2:**
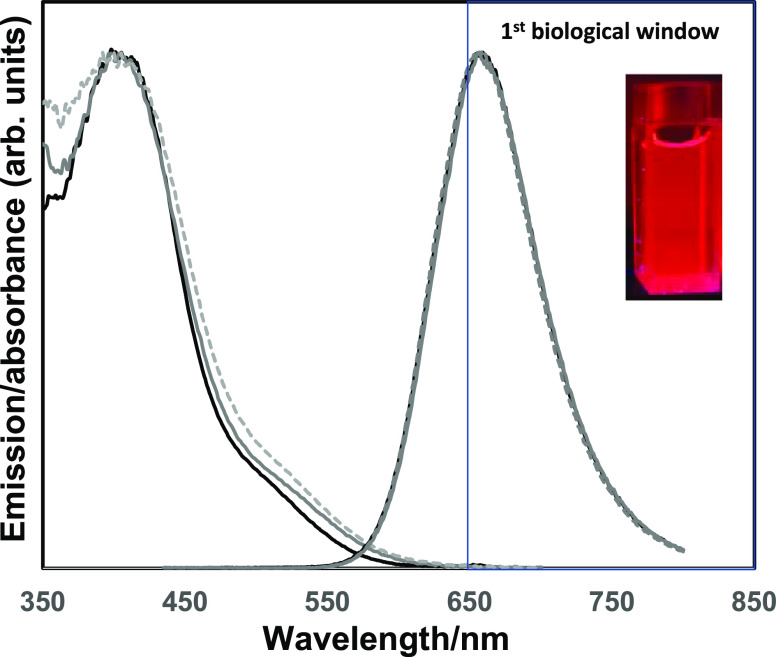
Normalized absorption (left hand side) and emission (right hand side) spectra of CN-FO-DPD in THF (solid black line), of the nanoparticles without SPIONs (solid gray line), and of the nanoparticles with SPIONs (dotted gray line). Inset: photograph of CN-FO-DPD/PSMA nanoparticles without SPIONs in H_2_O. λ_exc._ = 420 nm.

Figure 2 presents the normalized spectra of the CN-FO-DPD/PSMA nanoparticles (with and without SPIONs), against pristine CN-FO-DPD in solvent. The absorption spectra of the nanoparticles displayed a slight redshift in the onset of the absorption edge upon nanoparticle formation, which was more pronounced with the formation of nanoparticles that contained iron oxide. The iron oxide particle did not appear to contribute to the absorption spectra of the final particles. The redshifts in the optical properties upon processing conjugated polymers into thin films or particles^37−39^ were not observed, with the free conjugated polymer in solution and nanoparticle solution exhibiting an emission maximum at *ca*. 660 nm. This was attributed to the polymer not changing confirmation when in the nanoparticle state.

Changing the concentration of the conjugated polymer did not show any significant change in the emission profile of the NPs, although at 100 μg/mL, a broadening of the absorption spectra was observed (Figure S2A). Changing the ratio of conjugated polymer to PSMA again resulted in no significant change in the emission spectra of the NPs (Figure S3), although a broadening in the absorption spectra was observed, which may be due to conjugated polymer encapsulation in the varying amount of PSMA (Figure S3A,C). Fluorescence quantum yields (QY) of polymer and particles were calculated when excited at 370 nm using an integrating sphere, with the polymer in THF alone exhibiting a QY of 60%, which dropped to 32% upon being processed into colloidally stable nanoparticles. The inclusion of iron oxide into the particles reduced this further to 21%. This decrease in QY is likely due to aggregation-induced fluorescence quenching between the CN-FO-DPD and the SPIONs, which has been observed by Howes et al. and Feng et al.^33,40^

### Physical Characterization of Conjugated Polymer Nanoparticles

The diameter of the particles was determined using dynamic light scattering (DLS). Both the concentration of conjugated polymer and the amount of SPIONs added had some effect on the diameter of the NPs; as the amount of conjugated polymer (without SPIONs) was increased, an increase in the size of the NP was also observed, although the final diameter was still under 100 nm for particles prepared without iron oxide (Figure S4A). To determine if the SPIONs affected the size, varying volumes of SPIONs were added during the initial nanoparticle synthesis step, and it was seen that increasing the amount of SPIONs resulted in a general trend toward a larger overall diameter for the final particles. The NPs without SPIONs varied in diameter from 50 to 300 nm (average of 68 ± 13 nm), which increased upon inclusion of the iron oxide to an average diameter of 120 ± 19 nm.

The particles had a relatively narrow diameter distribution with narrow polydispersity indices (PDIs), as measured by the intensity of the scattered light, for those with a conjugated polymer/PSMA ratio ranging from 10:1 to 1:10 (Figure S5C). Changing the ratio of CN-FO-DPD to PSMA also affected the size of the CPNs, with an increase in the size of NPs as the amount of PSMA was increased (Figure S5A). This was particularly the case when there was 20 or 40 times as much PSMA to CN-FO-DPD. In these samples, the samples were cloudy rather than clear and difficult to filter.

Transmission electron microscopy (TEM) revealed nanostructures, as shown in Figure S6, which aggregated when dried on TEM grids with varying sizes of nanoparticles. On average, the particles had a total diameter between 60 and 70 nm, with the SPIONs being about 5–10 nm. As PSMA and conjugated polymers are composed mostly of carbon, there was little contrast although SPIONs could be observed as black particles encapsulated inside the polymeric structures. The slight difference between the sizes of the particles observed on the TEM *vs* the DLS measurements was attributed to the hydrated size of the particles in water.^43^ The iron oxide particles appeared scattered throughout the body of the parent particles, with no specific site preference.

A study of the ζ potential suggested an overall negative surface charge of the particles, consistent with the carboxylate group. It was observed that increasing the ratio of PSMA to conjugated polymer decreased the ζ potential (from −15 to −30 mV), and thus the nanoparticles became more stable, particularly beyond 2:1 (Figure S5B). The increase in the mV value for 20× and 40× PSMA to conjugated polymer was also observed, which supported the suggestion of aggregating nanoparticles as seen in the increasing size of the particles.

A superconducting quantum interference device (SQUID) magnetometer was used to characterize the magnetic behavior of the CPNs containing SPIONs (Figure S4D). The M–H curve of the SPIONs on their own exhibited saturation of magnetization (emu/g) up to 55 emu g^–1^ as previously observed.^41,42^ For CPNs with a CP/PSMA ratio of 2:1, we observed saturation of magnetization up to 20 emu g^–1^, which supported the observed superparamagnetic properties, which were further confirmed by the zero-net magnetization of the particle assemblies in the absence of an external field. Changing the ratio of the CP/PSMA influenced the moment of the CPNs, with a decrease in value from 20 to 2 emu g^–1^ and 1 emu g^–1^.

### Cellular Uptake and Imaging Studies

**Figure 3:**
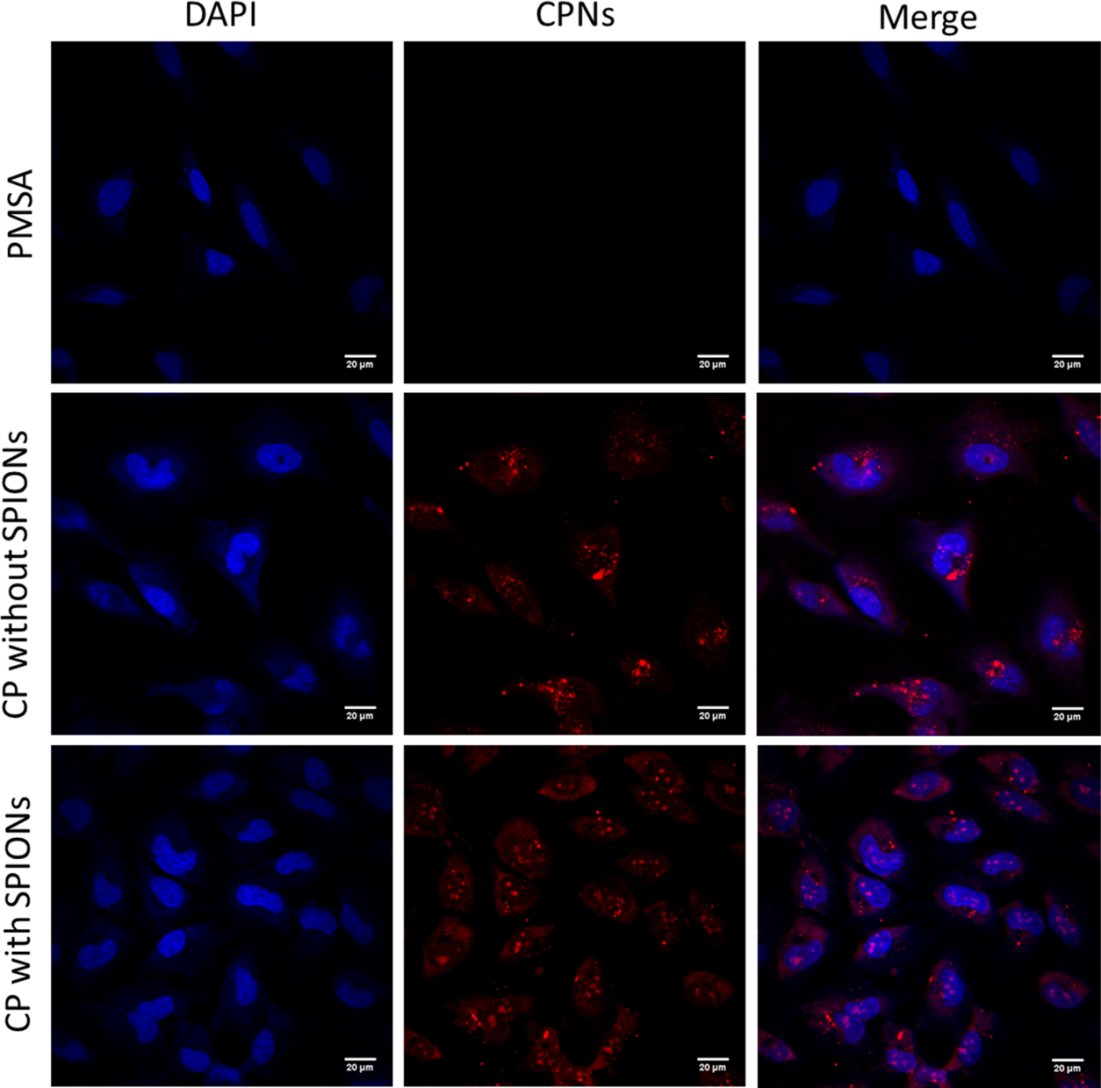
Representative epifluorescence images of HeLa cells (fixed and stained with DAPI (blue) to visualize DNA) 24 h after nanoparticle treatment (Red). Scale bar = 20 μm.

To investigate their potential use in biological imaging, conjugated polymer nanoparticles capped with PSMA (in a ratio of 2:1 conjugated polymer/PSMA, with and without SPIONs) were initially incubated with HeLa cells at a CN-FO-DPD concentration of 5 μg/mL for 24 h. After incubation, the cells were fixed and stained with DAPI for 30 min before imaging (Figure 3). The CPNs were bright with clear, defined red emission. Both CPNs with and without SPIONs appeared to be readily taken up by the cells, where they were uniformly distributed through the cytoplasm. While it was also clear that no nanoparticles were found within the nucleus, which is likely due to the size of the NPs, there appeared to be clusters of much brighter regions, which may have been cellular compartments and organelles.

**Figure 4:**
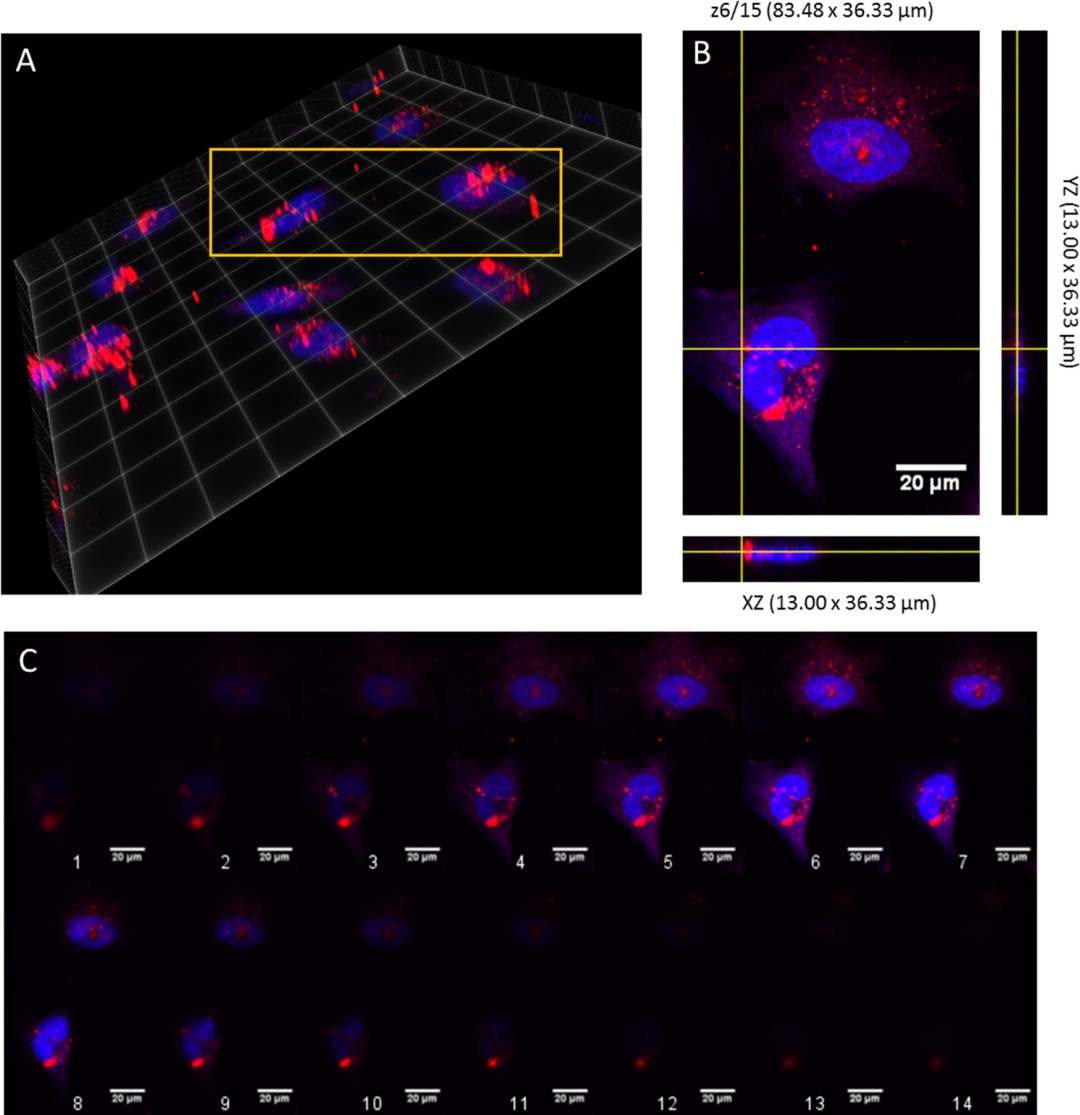
Z-Stack through a HeLa cell after 24 h of incubation with NPs without SPIONs at 0.32 μm steps. Red indicates NPs, whereas blue is DAPI stain. (A) Volume view at maximum intensity, with yellow box indicating area of interest. (B) Orthogonal view of z-stack, with slice 6/15 taken as showing NP internalized within the cell. (C) Montage of the Z-stack, with 1, indicating bottom of cell, to 14, indicating top (depth ∼10.45 μm). Scale bar = 20 μm.

To determine whether the nanoparticles were internalized into the cells, 15 optical sections through individual cells were imaged in incremental steps of 0.5 μm (total depth = 10.54 μm) from regions around the upper surface of the cell to the lower surface (Figure 3). It appeared from Figure 4C (6th slice) that the NPs were in the same plane as the stained nucleus (blue DAPI stain), thus being internalized. From both images, the polymer nanoparticles did not appear to be causing the cells any cytotoxic effects.

### Fluorescence Lifetime Studies of Conjugated Polymers in Dispersion and in Cells

For the fluorescence decay of the nanoparticles in the dispersed state, excited at 467 nm (Figure S7), we found by judging the residuals and the χ_R_^2^ that a triple-exponential model best fits the fluorescence decay. The three fluorescence lifetimes were: 0.7, 2.9, and 7.2 ns, where their amplitude contributions were given by 47.98, 39.22, and 12.80%, respectively. The average lifetime had a value of 4.3 ns, which appears to be within the range of other observed conjugated polymers.^44^

**Figure 5:**
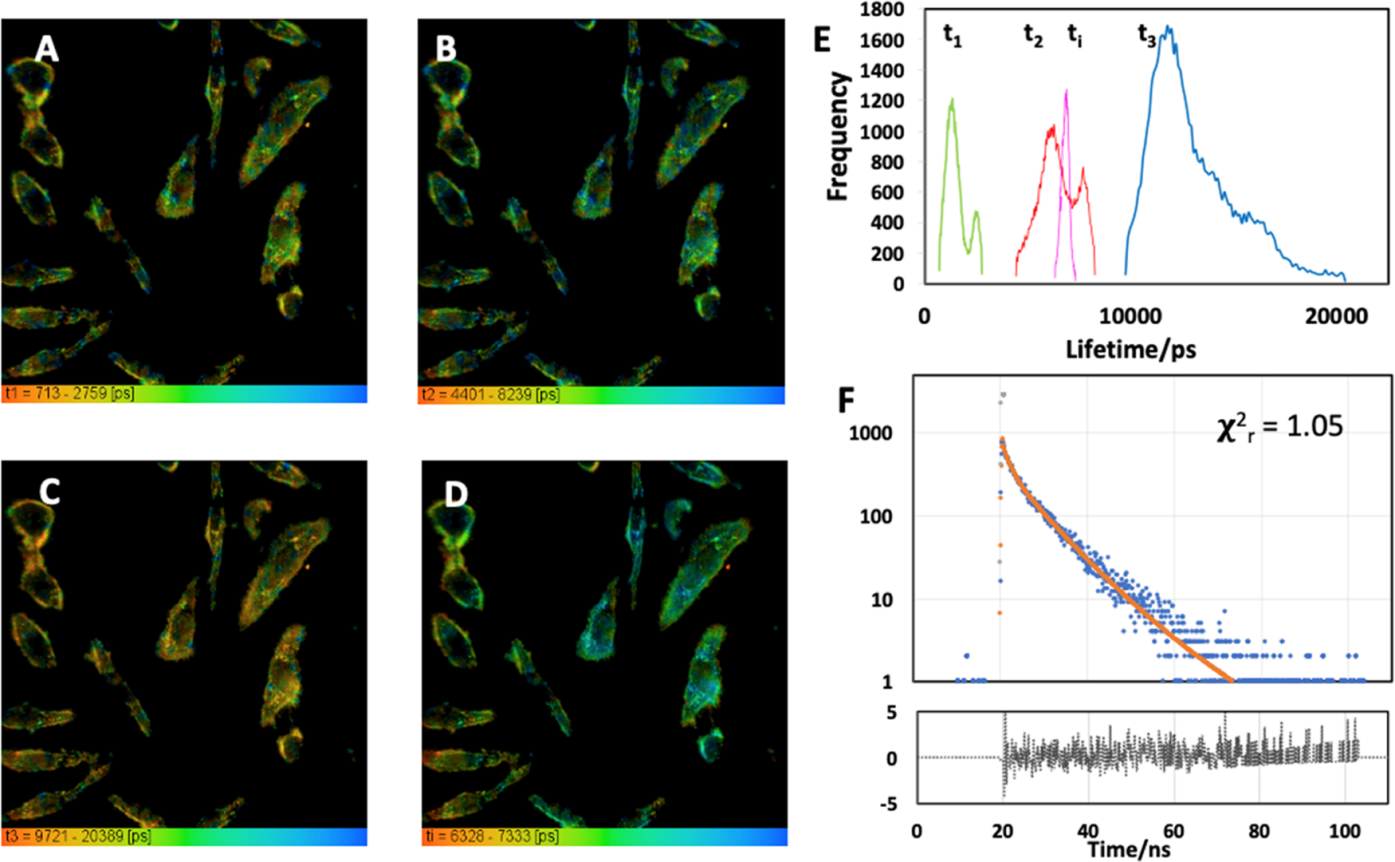
FLIM of the CN-FO-DPD (with SPIONs) nanoparticles in live cells at 37 °C (5% CO_2_), where (A–C) corresponds to each one of the fluorescence lifetimes which each fluorescence decay has been fitted with (*A* = *t*_1_; *B* = *t*_2_; *C* = *t*_3_). (D) Average fluorescence lifetime (*t*_i_). (E) Histogram for each one of the fluorescence lifetime components along the average lifetime and (F) representative fluorescence decay (blue) for this image, the instrument response (gray), the fitting (red), and the residuals (black). The residuals are flat, and χ_R_^2^ = 1.05 indicates a good fit. The images are 238 μm along each side.

For fluorescence lifetime imaging (FLIM) microscopy, HeLa cells were incubated with the nanoparticles. They were found to be distributed mainly in the cytoplasmic region of the cells as shown earlier by epifluorescence microscopy, as shown in Figure 5. We found that for the nanoparticles in HeLa cells, a triple-exponential decay model also best fits the data. Compared to the cell-free results, particles in cells exhibited longer lifetime values but similar amplitude contributions. The three lifetimes were given as 1.3, 6.5, and 12.1 ns, where their amplitude contributions were 50.34, 41.73, and 7.93%, respectively. These values yield a mean lifetime of 7.0 ns, so the fluorescence lifetime of the nanoparticles increased compared to the native dispersion when internalized in cells.

### Cytotoxicity Experiments

We evaluated the cytotoxicity of the nanoparticles by measuring the viability of human embryonic kidney cells 293 (HEK293) using a CellTiter-Glo luminescent cell viability assay (Promega, U.K.), which determined the number of viable cells in cultures based on quantitation of the ATP present, to give an indication of metabolically active cells. The greater the number of metabolically active cells, the greater the levels of ATP, which was represented as luminescence. These were normalized against the control containing no particles, and with both sets of CPNs, there was no significant difference in the luminescence values in HEK cells up to 48 h, which suggested no inherent toxicity occurred from the CPNs. However, the amount of luminescence decreased at 48 h (Figure S8) in all samples, including the negative control cells, which could be due to the cells reaching maximum confluency at 24 h and no longer proliferating, meaning a decrease in the production of ATP.^45^ Bare CN-FO-DPD particles (without PSMA or SPIONs) were persistently stable, showing no change in size over time, and did not appear to affect cell viability.

**Figure 6:**
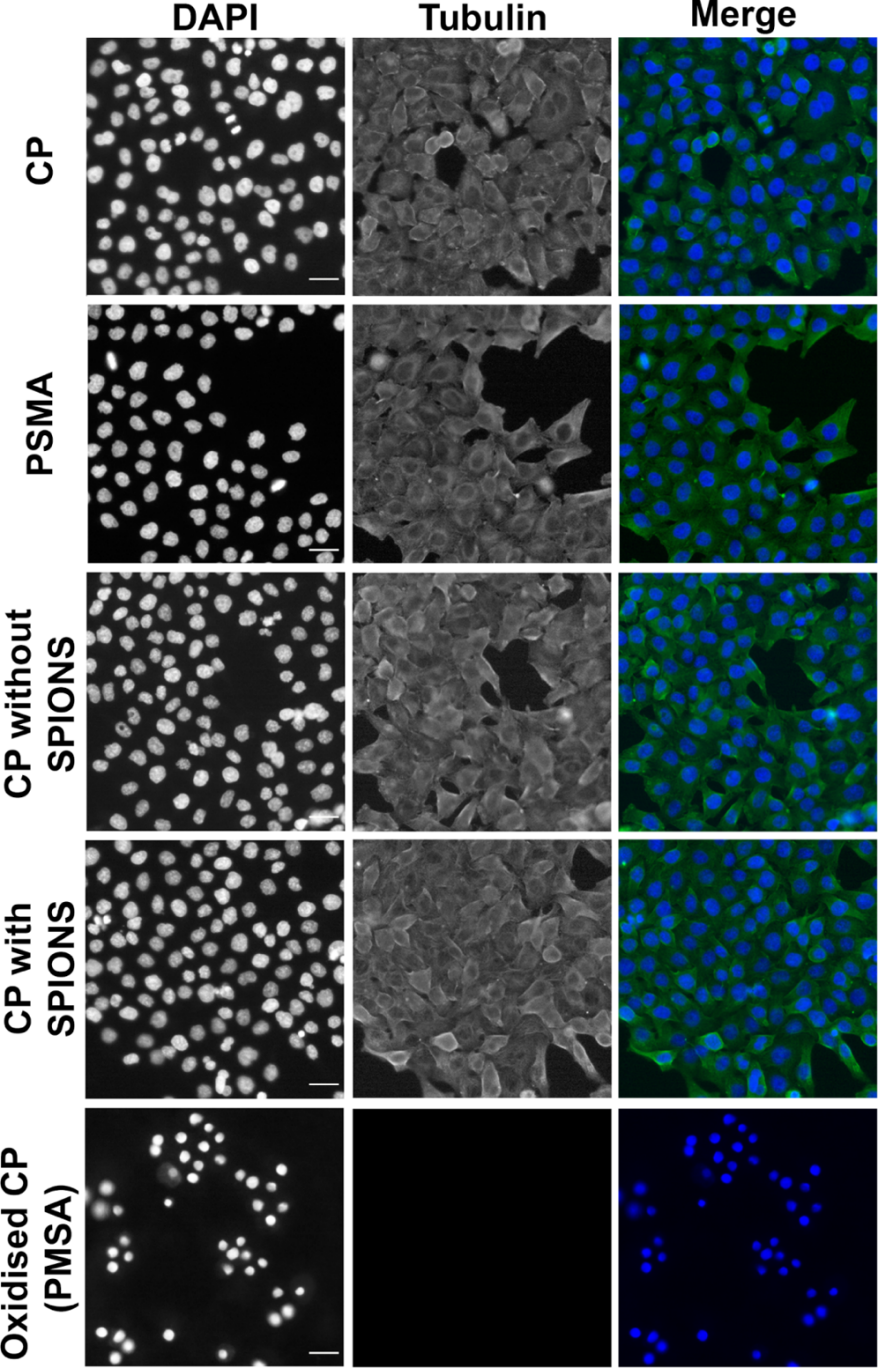
HeLa cells do not show any mitotic arrest or defects after conjugated polymer particle treatment (7.5 μg/mL) (without SPIONs) and a total solid concentration of 50 μg/mL (with SPIONs). Representative epifluorescence images of HeLa cells fixed and stained with anti-αTubulin (green) and DAPI (blue) to visualize Tubulin and DNA, respectively, 24 h after nanoparticle treatment. Conjugated polymer nanoparticles (no PSMA) and PMSA nanoparticles are negative controls, and oxidized conjugated polymer nanoparticle is a positive control known to be toxic. Scale bar = 50 μm.

The cytotoxicity of the conjugated polymer nanoparticles was further tested by the analysis of the proliferation of HeLa cells using live cell imaging to estimate the potential effects caused by 24 h exposure to the NPs (at concentrations of 0.01, 0.1, and 1 μg/mL). HeLa cells did not show any evident toxicity related to proliferation compared to a positive control, (oxidized CPNs which were prepared by the procedure in ref (46), in which the conjugated polymer MEH-PPV was oxidized with H_2_O_2_ and then encapsulated in PSMA as shown in Figure S9). Following the examination of live cells, we studied the cytoskeletal protein tubulin and DNA in fixed HeLa (Figure 6) and HCE, a noncancerous cell line (Figure S9). Unlike in the positive control, there was no evidence of DNA fragmentation or mitotic arrest in cells treated with nanoparticles, further supporting our findings that the nanoparticles reported here do not adversely affect cells.

### Activation of Apoptosis

After determining that the nanoparticles did not induce obvious cell death or DNA fragmentation in live and fixed cells (7.5 μg/mL (without SPIONs)) and a total solid concentration of 50 μg/mL (with SPIONs), we directly tested the activation of the apoptotic pathway by the particles. We quantified the presence of Annexin V,^47^ a common marker of apoptosis. This protein binds phosphatidylserine (PS) during early apoptosis where PS becomes exposed at the cell surface several hours before DNA fragmentation can be detected.^48,49^ No significant activation of the early apoptotic pathway (Annexin V-FITC positive) (data are presented as mean +/– SEM (*n* = 3), >350 cells were scored per experiment (****p < 0.001)) was observed in HeLa or HCE treated with CN-FO-DPD/PSMA nanoparticles (with and without SPIONs). In contrast, all cells were Annexin V-FITC-positive in cells treated with oxidized conjugated polymer NP nanoparticles (Figures S10 and S11).

### *In Vivo* Study of CPNs in Zebrafish

**Figure 7:**
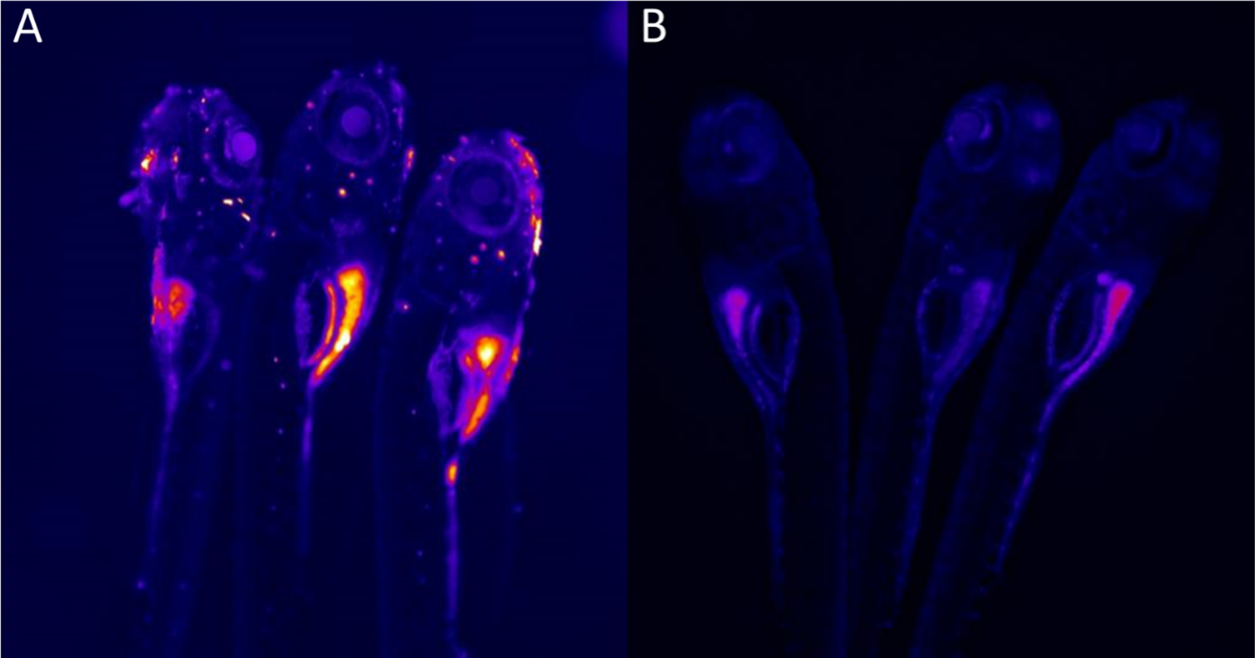
Fluorescence image of day 1 zebrafish embryos treated with (A) CN-FO-DPD/PSMA (2:1) nanoparticles (without SPIONs) *vs* (B) untreated. These were excited with a 488 nm laser.

As a further technique to highlight the suitability of the conjugated polymer particles for biological imaging, zebrafish larvae were treated with 50 μg/mL CN-FO-PDP:PSMA nanoparticle dispersions (5 mL total one volume) and allowed to grow in water mixed with the sample. In Figure 7A, it can be seen that in one-day-old embryos, the nanoparticles seem to be most prevalent within the gut of the embryo as expected. Compared to the zebrafish that are not treated, it was difficult to determine where the CPNs have co-localized, considering that they are not surface-functionalized. However, in the following days, the nanoparticles appear only on the outer skin (Figure S12B,D). Despite this, the CPNs do not appear to be cytotoxic to the embryos, with larvae growing within CPN solutions for 5 days with no changes in morphology or increased mortality.

## Conclusions

In conclusion, we present the use of a biocompatible red-emitting conjugated polymer particle system for use in cellular imaging. The particles were colloidally stable and exhibited high fluorescence quantum yields, even when incorporated with iron oxide (21%). Both the absorption and emission spectra of the polymer did not change position with the formation of the particles or the addition of SPIONs, which allowed for a cheaper, simpler purification of the nanoparticles through a magnetic pulldown. The particles that contained SPIONs exhibited magnetism ranging from 2 to 20 emu/g depending on the amount of PSMA capping agent. We also showed that these particles were capable of being endocytosed by different cell types, remaining bright within the cells while Z-stacks confirmed that the NPs were located within the cells, and not on the cell surface. We finally showed that the particles were not cytotoxic, using both Annexin V and CellTiter-Glo luminescent cell viability assays. The NPs have the potential for use in further biological imaging: both as a means for target-specific binding when modified and functionalized and as a potential multimodal tool with the addition of SPIONs.

## Methods

### Materials

Poly[{9,9-dihexyl-2,7-bis(1-cyanovinylene)fluorenylene}-alt-co-{2,5-bis(*N*,*N*′-diphenylamino)-1,4-phenylene}] (CN-FO-DPD) (MW 40,000–70,000) was purchased from American Dye Source, Inc. (Canada). Poly(styrene-*co*-maleic anhydride), cumene-terminated (PSMA) (MW 1700), tetrahydrofuran (THF), magnetic iron oxide nanoparticles (10–40 nm, 0.8–1.4% total solid in heptane) (SPIONs), and fetal bovine serum (FBS) were purchased from Sigma-Aldrich (England, U.K). CellTiter-Glo reagent was purchased from Promega (England, U.K). All materials were used as received.

### Preparation of Conjugated Polymer Nanoparticles

#### CN-FO-DPD Nanoparticles

The method was adapted from Wu et al.^35^ CN-FO-DPD (10 mg) and PSMA (10 mg) were added to separate vials and dissolved in 10 mL of THF (1 mg/mL) followed by sonication for 10 min. CN-FO-DPD stock (0.5 mL) and PSMA stock (0.25 mL) were added to 0.25 mL of THF. The solution was then injected into 9 mL of deionized water and sonicated for 10 min. The resulting dispersion was then stirred continuously at 400 rpm, at room temperature, for 24 h to evaporate off THF. The loss of water was compensated by readjustment to 10 mL. The nanosuspension (50 μg/mL of CN-FO-DPD or total solid of 0.075 mg/mL) was subsequently filtered through a 0.2 μm cellulose acetate Gilson syringe filter. The filtrate was stored at room temperature.

#### CN-FO-DPD/Iron Oxide Nanoparticles

As above, 0.5 mL of CN-FO-DPD stock and 0.25 mL of PSMA stock were added to 0.20 mL of THF. The iron oxide nanoparticles dispersion (5 mL, 11.7–20 mg/mL) was taken, and the heptane was removed before being resuspended in 1 mL of THF (52–89 mg/mL). Of this, 0.05 mL was added to the THF solution containing CN-FO-DPD and PSMA to give a total volume of 1 mL. The mixture was then injected into 9 mL of deionized water and sonicated for 10 min. The dispersion was then stirred continuously at 400 rpm, at room temperature, for 24 h to evaporate off THF. Loss of water was compensated by readjustment to 10 mL. The nanosuspension (50 μg/mL of CN-FO-DPD/iron oxide or total solid 0.335–0.52 mg/mL) was placed against a neodymium magnet with a pull force of 10 kg for 24 h. Nonmagnetized NPs were removed, and the particles were resuspended in 1 mL of deionized H_2_O and stored at room temperature.

#### Oxidized Conjugated Polymer Nanoparticles

Oxidized conjugated polymer nanoparticles were prepared as in previous work.^46^ MEH-PPV (10 mg) was added to 10 ml of THF and left stirring overnight to ensure complete dissolution of MEH-PPV. To this, 10 ml of H_2_O_2_ (30%) was added to 10 mL of THF and a serial dilution was made to 1% H_2_O_2._ To this, 0.5 mL of the MEH-PPV in THF was added and left for 1 week. The oxidized MEH-PPV solution (1.5 mL, 0.05 mg/mL) was added to 1.5 mL of THF containing 0.03 mg of PSMA. The solution was sonicated in a 35 kHz ultrasound bath at 7–9 °C, in 30 s bursts for 5 min to ensure all polymers were completely dissolved. The solution was then injected into 5 mL of deionized water and sonicated for 10 min. The solution was then stirred continuously at 400 rpm, at room temperature, for 24 h to evaporate off THF. Loss of water was compensated by readjustment to 5 mL. This was further dialyzed in a 50k Da Spectra/Por Biotech Cellulose Ester (CE) membranes (Spectrum Chemical, USA) for 24 h to remove residual H_2_O_2_. The nanosuspension (10 μg/mL MEH-PPV or total solid of 0.015 mg/mL) was subsequently filtered through a 0.2 μm cellulose acetate Gilson syringe filter and stored at room temperature.

#### CPNs Optical and Physical Measurements

Absorption spectra were measured using a Hitachi U-4100 UV–visible-NIR spectrometer using a 1 cm pathlength quartz cuvette. Fluorescence spectra were measured using a Horiba Fluoromax-4 spectrofluorometer. Particle size distributions and ζ potentials were obtained using a Malvern Zetasizer (utilizing dynamic light scattering). Transmission electron microscopy images were acquired on a Hitachi 7100 at St George’s University of London, with a filament electron source at 100 kV. Image analysis was performed with ImageJ software. The absolute QY was measured using 370 nm excitation from a diode Thorlabs laser, utilizing a Newport SS 6″ integrating sphere, and recorded using an ocean optics HR4000CG-UV-NIR spectrometer. The system response was calibrated using ocean optics calibrated white light source. Magnetic measurements were done at the London Centre of Nanotechnology using a physical property measurement system (PPMS, Quantum design): 3.4 mg of the SPIONs, 2.1 mg of 2:1 CP/PSMA, 1.1 mg of 1:5 CP/PSMA, and 0.5 mg of 1:10 CP/PSMA were weighed, and the applied field ranged from −6 to 6 kOe at 310 K.

#### Cell Culture

HeLa cells used in the Eggert group (Figures 5 and SI 8, 9, and 10) were verified as HeLa by STR profiling from Eurofins MWG. HCEs were a gift from Min S. Chang (Vanderbilt University, Nashville, Tennessee).^50^ HEK cells were provided by the Zammit Group.^51^ HeLa, HCE, and HEK cells were grown at 37 °C in complete DMEM (Invitrogen) supplemented with 10% fetal bovine serum (FBS) and 1% penicillin-streptomycin in T75 tissue culture flask (Helena TTP).

#### Nanoparticle Treatment

HeLa and HCE cells were grown on a 24-well plate (ibidi) overnight at 30,000 cells/mL, and HEK cells were cultured in a 96-well plate in subconfluency 24 h prior to experiments. The cells were treated with NPs, negative and positive controls for 24 h. The CN-FO-DPD nanoparticle suspension was diluted in DMEM to have a total solid concentration of 7.5 μg/mL (without SPIONs) and a total solid concentration of 50 μg/mL (with SPIONs). The CN-FO-DPD nanoparticle suspension (500 μL) was added to 500 μL of the media (for the 24-well plate), and the CN-FO-DPD nanoparticle suspension (20 μL) was added to 100 μL of the media (for the 96-well plate). These were incubated for 24 h prior to fixation for immunofluorescence or live cell imaging.

#### Immunofluorescence

Cells were fixed with 4% paraformaldehyde in phosphate-buffered saline (PBS) (Sigma-Aldrich) for 20 min at room temperature; cells were permeabilized by incubation with 0.3% Triton X-100 in blocking buffer (0.5% bovine serum albumin, 0.1% NaN_3_ in PBS) for 5 min followed by three 5 min incubations in blocking buffer plus 20 mM glycine. Plates were incubated with primary antibodies, diluted in blocking buffer at 4 °C overnight, washed three times (5 min/wash) with blocking buffer, and incubated with the appropriate secondary antibodies and 4′,6-diamidine-2′-phenylindole at 1 μL/mL (DAPI) (Cell Signalling Technologies, Inc.) for 45 min at room temperature. The plates were washed three further times with blocking buffer.

#### Antibodies

Primary antibody anti-α-tubulin (Sigma: T9026) was utilized. All appropriate secondary fluorescent conjugated antibodies were purchased from Jackson ImmunoResearch.

#### Apoptosis Assessment

The evaluation of nanoparticle-induced apoptosis was evaluated by Annexin V staining and CellTiter-Glo assay. For the Annexin V assay, cells were grown on a 24-well plate (ibidi) overnight at 30,000 cells/mL. After the cells were treated with NPs (total solid concentration of 7.5 μg/mL (without SPIONs) and a total solid concentration of 50 μg/mL (with SPIONs)), negative and positive controls for 24 h, the cells were washed with cold PBS and incubated with Annexin V solution containing 1/100 dilution of Annexin V-FITC in Annexin V binding buffer (TACS Annexin V-FITC Kit, Trevigen) for 15 min at room temperature in the dark. The cells were washed twice with 1× binding buffer at room temperature and fixed as shown before. For the cytotoxicity study with HEK cells, after the time intervals of 1, 24, and 48 h, 100 μL of CellTiter-Glo reagent was added and the cells were left to lyse for 10 min before the luminescence was recorded.

#### Zebrafish

Zebrafish (AB strain) were raised and maintained using standard husbandry conditions^52^ in accordance with U.K. Home Office regulations. Larval zebrafish at 4 days post-fertilization were treated with nanoparticles at a total solid concentration of 7.5 μg/mL nanoparticles in embryo media (E3). After 2 h incubation at 28 °C, the nanoparticles were washed out with fresh E3 media. The larvae were anesthetized with tricaine, immobilized with agarose, and imaged with a Leica SPE confocal microscope. The larvae were excited at 405 nm and collected at ca. 510–735 nm. The larvae were also exposed to this concentration for 24 h to test their tolerance to the solution. No changes in behavior or development were observed.

#### Image Acquisition

FLIM measurements were taken with a TCSPC (time-correlated single photon counting) card in combination with an inverted confocal microscope (Leica TCS SP2). The system was excited with a picosecond diode laser (Hamamatsu PLP-10 470) at 467 nm and a repetition rate of 10 MHz. The fluorescence emission was detected by a GaAsP hybrid detector (Becker & Hickl HPM-100-40, based on a Hamamatsu R10467-40 GaAsP hybrid photomultiplier) prior to collection by the TCSPC module (SPC-150) and the excitation light was discriminated from the emission using a 500LP filter. An RSP 500 excitation beam splitter and a 63 × 1.2 N.A. water-immersion objective were used to acquire the images. The line scan speed was set to 400 Hz, and the image size was set to 512 × 512 pixels with a pixel size of 470 × 470 nm^2^ and a pinhole of 2 Airy units. The laser power was on average a few μW. The collected data were analyzed with SPCImage (Becker & Hickl).

For analysis, fixed cells were imaged using inverted Nikon Eclipse microscopes using wide-field epifluorescence (Ti-E) equipped with a Cool SNAP HQ 2, DS-Fi2 Color CCD camera using 20× and 40× air and 60x oil objectives. Movies were recorded on a Nikon Ti-Eclipse microscope with an environmental chamber to maintain cells at 37 °C and 5% CO_2_ using a 20× air objective. Movies were recorded at 1 frame every 10 min, and cells were incubated in FluoroBright DMEM (Invitrogen) supplemented with 10% fetal bovine serum (FBS, Sigma), 1% penicillin-streptomycin (PenStrep, Invitrogen), and 1× l-glutamine and 10 mM sodium pyruvate (Life Technologies). Analysis of the area covered by the cells in the 24 h time lapse was calculated using ImageJ software. Briefly, the ratio of free space (areas without cells) to the whole image field (remains constant when using the same imaging setting) was measured at time 0 and 24 h, so a comparison of the two time points was possible. Original data were processed using NIS elements software (Nikon), ImageJ, and Adobe Photoshop CS5.1. Images were generated using ClearVolume^53^ and ImageJ 3D viewer.

#### Statistical Analysis

For spectra, quantum yields and size values from DLS all experiments were determined in triplicate with the data presented as average. For counts of multinucleated cells, mean and 1 standard deviation from the mean were calculated for *N* ≥ 2 independent experiments unless otherwise stated (typically > 200 cells were counted for each data point experiment unless otherwise stated). Bar graphs were drawn using Origin or GraphPad Prism. Statistical significance was assessed using an unpaired two-tailed Student’s *t*-test assuming unequal variance.

## Supporting Information Available

PL intensity over 30 days, optical properties at different concentrations, with and without SPIONs, graphs of concentration versus size, reagent ratio *vs* size/ζ potential/PDI, magnetic measurements, electron microscope images, fluorescence decay curves, emission in cells, and microscope images of CPNs in cells and in zebrafish (PDF)
