# Supplementary material for: Biocompatible Magnetic Conjugated Polymer Nanoparticles for Optical and Lifetime Imaging Applications in the First Biological Window
Source: ACS Appl Polym Mater. 2022 Oct 12;4(11):8193–202. doi: 10.1021/acsapm.2c01153 (PMC9667460; doi:10.1021/acsapm.2c01153)
Supplement: Supplementary file 1 — ap2c01153_si_001.pdf [file ap2c01153_si_001.pdf]

Supporting Information

Biocompatible magnetic conjugated polymer nanoparticles for optical and lifetime imaging applications in the first biological window.

Struan Bourke[a,f], Federico Donà[b,c], Yurema Teijeiro Gonzalez[a], Basma Qazi Chaudhry[a], Maryna Panamarova[b], Eirinn Mackay[d], Peter S. Zammit[b], Lea Ann Dailey[e], Ulrike S. Eggert[b], Klaus Suhling[a] and Mark A. Green.[*a]

[a] Department of Physics, King's College London, London, WC2R 2LS, UK. mark.a.green@kcl.ac.uk

[b] Randall Centre for Cell and Molecular Biophysics, Faculty of Life Sciences and Medicine, King's College London, London, SE1 1UL, UK.

[c] Current address; Department of Experimental Oncology, IEO, European Institute of Oncology, 20144, Milan, Italy.

[d] Department of Cell and Developmental Biology, University College London, Gower Street, London WC1E 6BT, UK.

[e] Department of Pharmaceutical Technology and Biopharmaceutics, University of Vienna, Universitätsring 1, 1010 Vienna, Austria.

[f] Current address; Department of Pharmaceutical Chemistry, University of California, San Francisco, San Francisco, CA, USA, 94158.

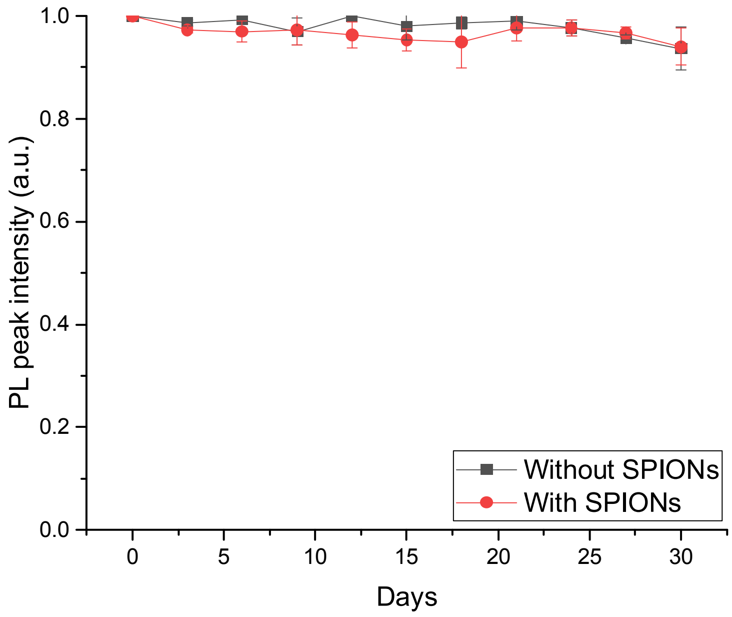

Figure S1. Normalised PL peak intensity values of CN-FO-DPD:PSMA nanoparticles ,with and without SPIONs, taken over 30 days. Values represent the mean of n = 3 replicate batches of nanoparticles.

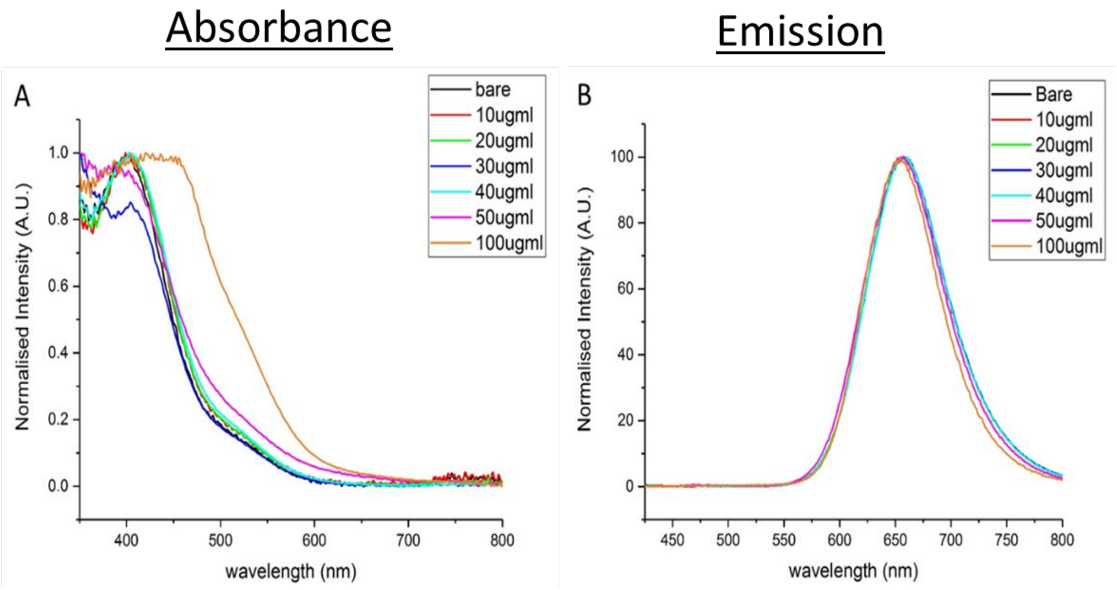

Figure S2. Normalised spectra of the different concentration of CN-FO-DPD in THF A) shows absorption of NPs and B) shows emission of NPs without SPIONs. Values represent the mean of n = 3 replicate batches of nanoparticles.

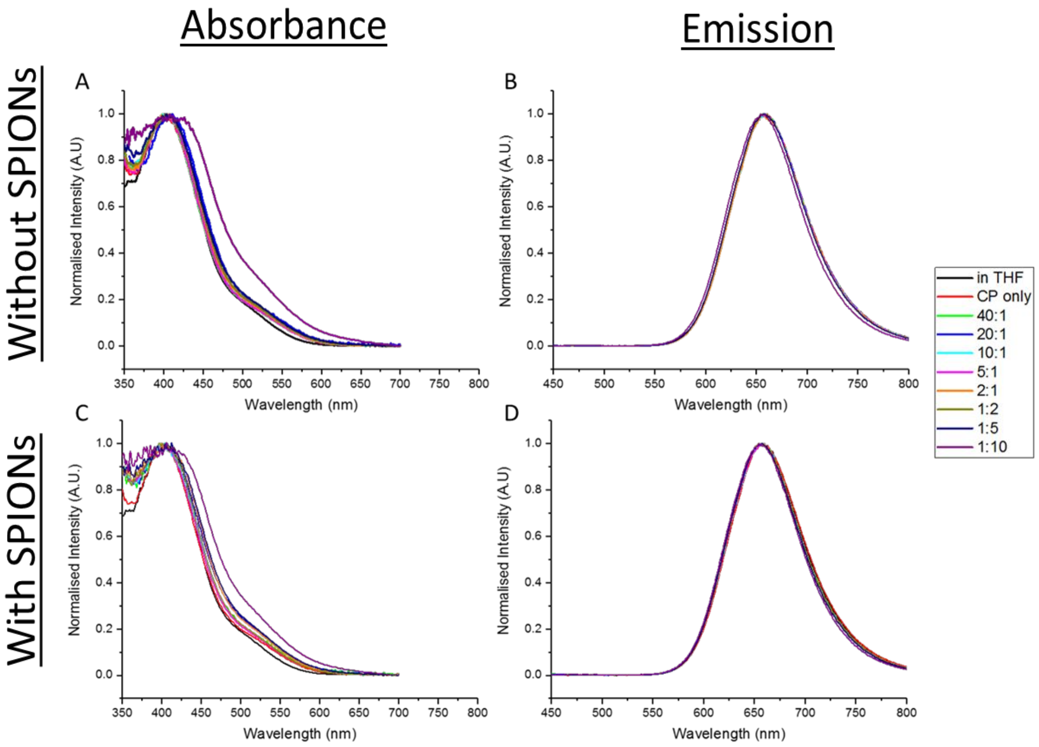

Figure S3. Normalised spectra of the different ratio of CN-FO-DPD to PSMA. A) shows absorption of nanoparticles without SPIONs, B) shows emission of nanoparticles without SPIONs. C) absorption of nanoparticles with SPIONs, D) shows emission of nanoparticles with SPIONs. λ_{abs} for the CN-FO-DPD was 420 nm, λ_{em} for the nanoparticles was 660 nm. Sample concentrations are 10 µg/ml. Values of spectra represent the mean of n = 3 replicate batches of nanoparticles.

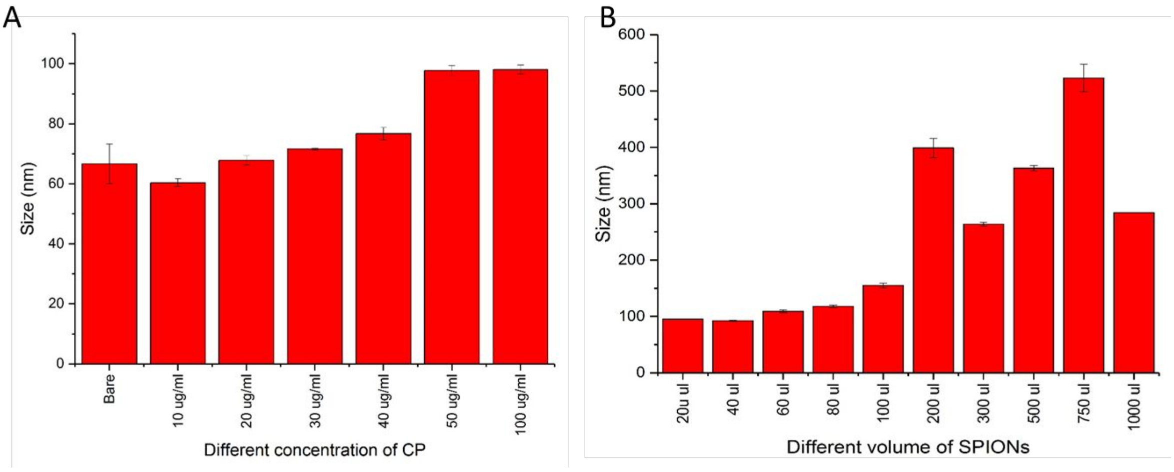

Figure S4. A) Graph of different concentration of conjugated polymer vs hydrodynamic diameter (nm) of the CPNs (n = 3). B) Graph of different volumes of SPIONs added vs hydrodynamic diameter (nm) of the CPNs. Values represent the mean ± standard deviation of n = 3 replicate batches of nanoparticles.

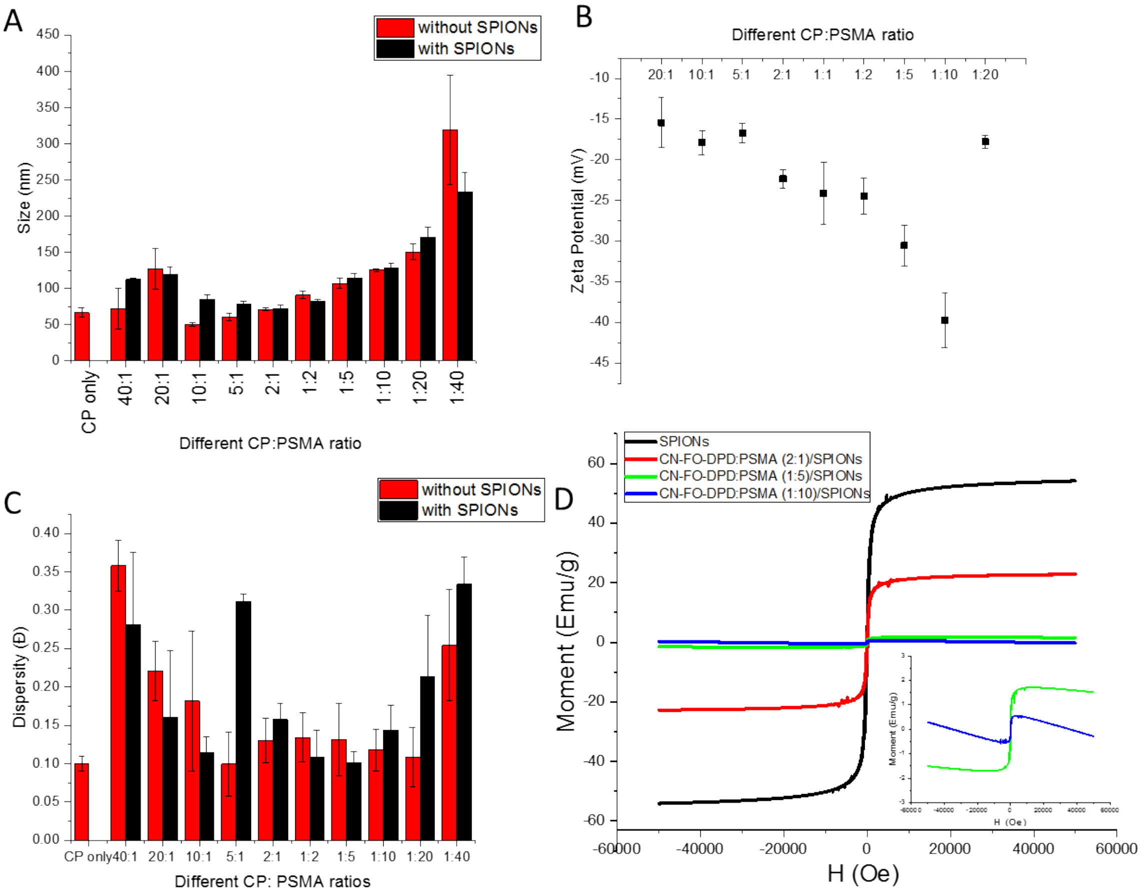

Figure S5. A) Graph of different CP:PSMA nanoparticle ratios vs mean diameter (nm) of the CPNs. Nanoparticles that do not contain SPIONs (red), Nanoparticles that contain SPIONs (black) (n = 3). B) Graph of different CP:PSMA ratios vs zeta potential (mV) of the CPNs not containing SPIONs (n=3). C) Graph of the different CP:PSMA ratio vs PDI. Nanoparticles that do not contain SPIONs (red), Nanoparticles that contain SPIONs (black) (n=3). D) Magnetic measurements from the SQUID magnetometer, showing the M-H loops at 310K of different CPNs. SPIONs (black) contain no PSMA or conjugated polymers, whereas CPNs at 2:1 conjugated polymer:PSMA ratio (red) have a decrease in emu g^{-1} value. Insert, shows both CPNs at 1:5 (green) and 1:10 (blue) conjugated polymer:PSMA ratio. Values represent the mean ± standard deviation of n = 3 replicate batches of nanoparticles.

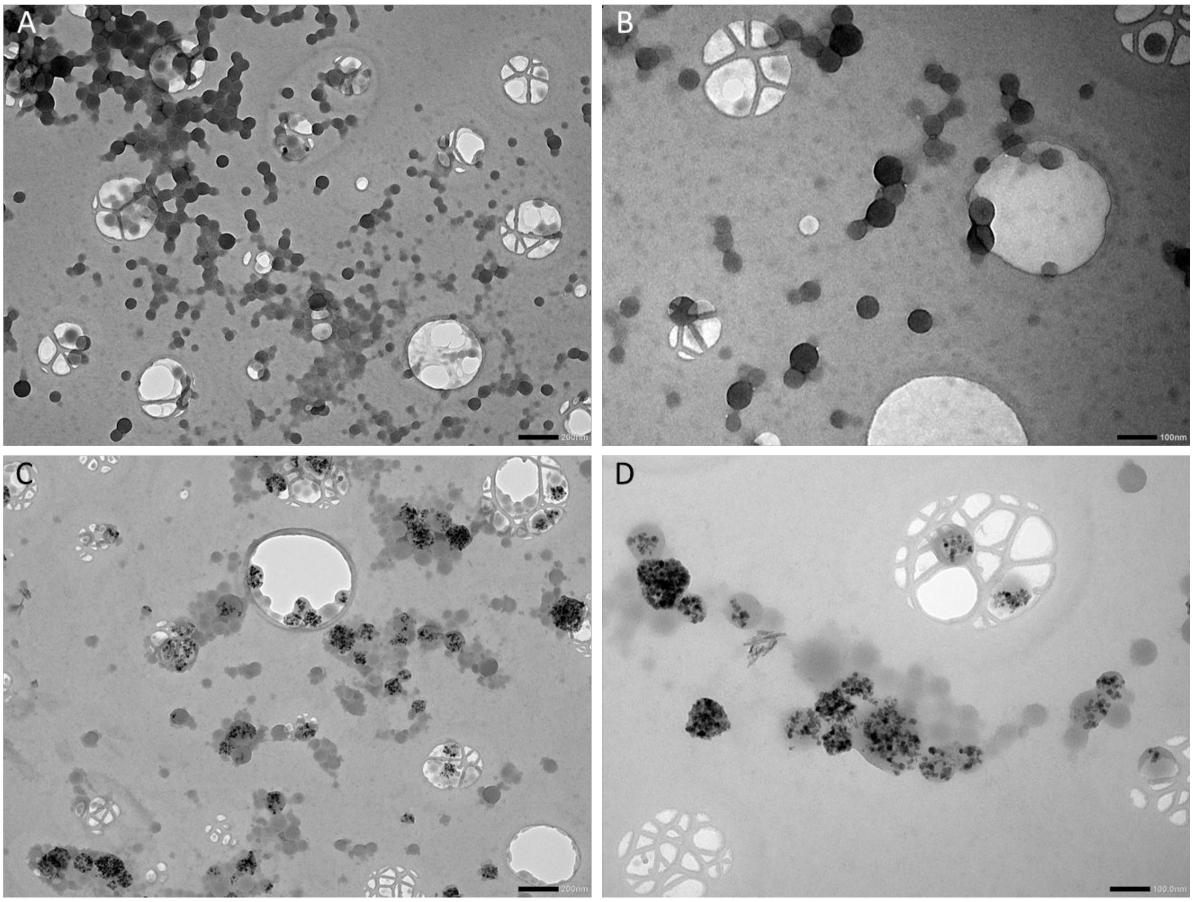

Figure S6. TEM images of the CN-FO-DPD:PSMA nanoparticles at a ratio of 2:1 (conjugated polymer to PSMA). A) and B) are CN-FO-DPD:PSMA nanoparticles without SPIONs, C) and D) are NPs with SPIONs, A) C) were taken at x20K (A & C scale bars = 200 nm, B & D scale bars = 100 nm).

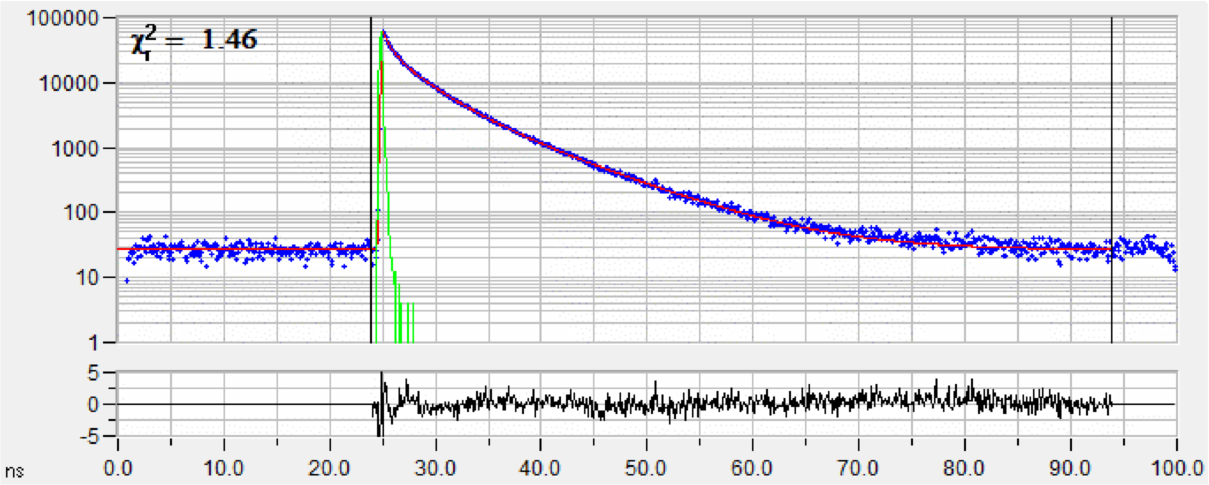

Figure S7. Fluorescence decay of the CN-FO-DPD (with SPIONs) nanoparticles in solvent, excited at 467 nm fitted to a triple-exponential decay model. The three fluorescence lifetimes were: 0.7, 2.9 and 7.2 ns, where their amplitude contributions were given by 47.98%, 39.22% and 12.80%, respectively. The average lifetime had a value of 4.3 ns. The fluorescence decay of the nanoparticles (blue) and the instrumental response (green) was measured by TCSPC. The fitting is in red and the residuals in black.

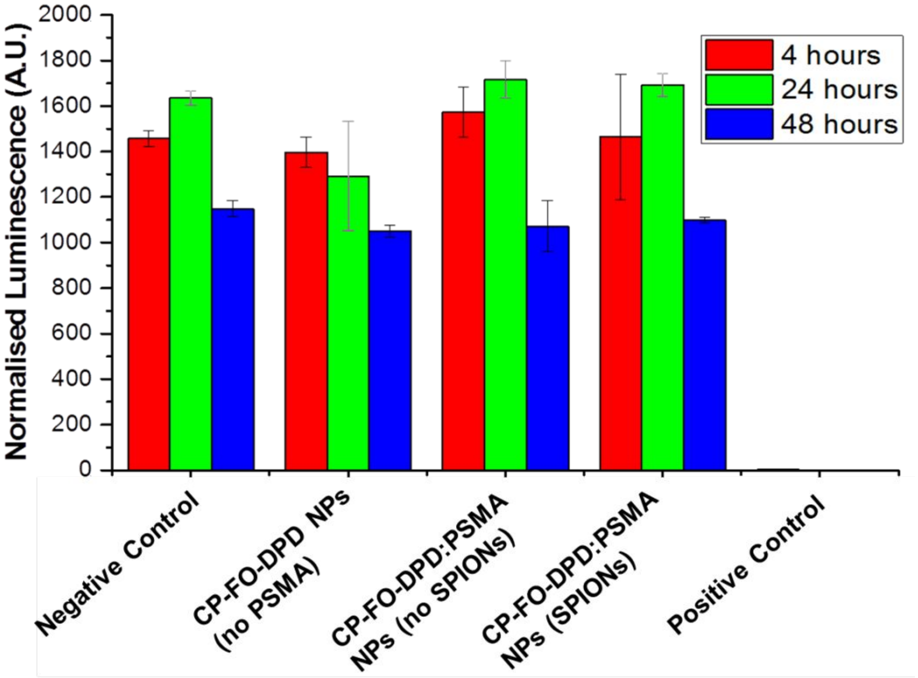

Figure S8. Normalised luminescence of negative control (just surfactant) vs with/without SPIONs vs positive control (oxidised CPNs which were prepared from [1], in which the conjugated polymer MEH-PPV was oxidised with H_2O_2 and then encapsulated in PSMA) incubated in HEK cells. These were normalised against control wells with no nanoparticles. Luminescence signal is related to level of ATP present in the cell, with higher levels of ATP present in metabolically active cells. Values represent the mean ± standard deviation of n = 3 replicate batches of nanoparticles.

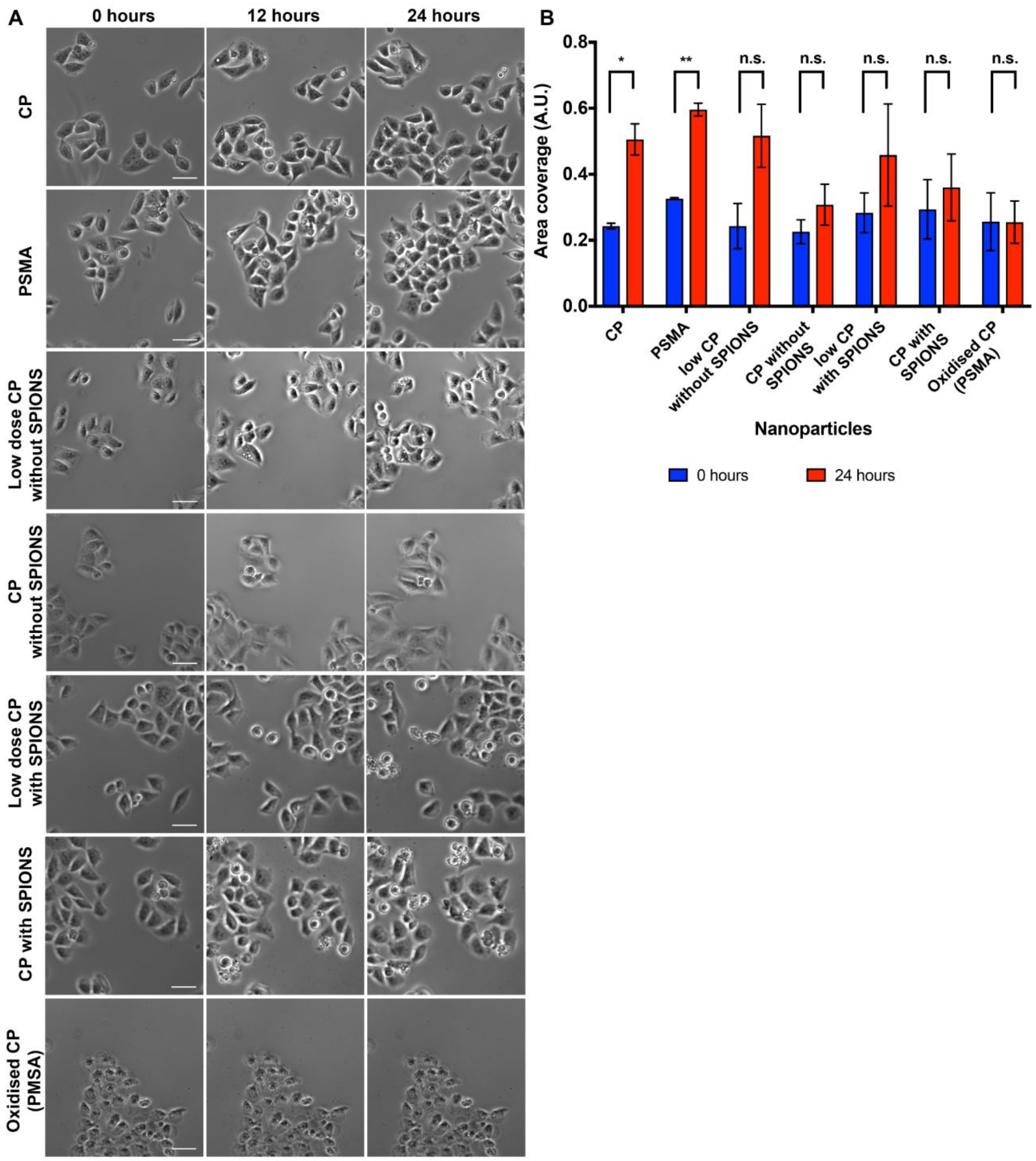

Figure S9. Conjugated polymer particles (CP) treatment does not perturb cell proliferation. (A) Still images from phase contrast time-lapse movies of HeLa cells treated with NPs for 24 h. Conjugated polymer nanoparticles (no PSMA) and PMSA nanoparticles are negative controls and the oxidised conjugated polymer particles were a known toxic positive control. Dose was 0.1 µg/ml. Time between frames is indicated in hours, and acquisition starts 15 min after CP treatment. Scale bar = 20µm. (See supporting information movies S1-7). (B) Quantification of the area covered by the proliferating cells at the starting point (0 h) compared to the end point (24 h). Data are presented as mean +/- S.E.M (n=2) (n.s. = non-significant, * p<0.05, **p<0.005).

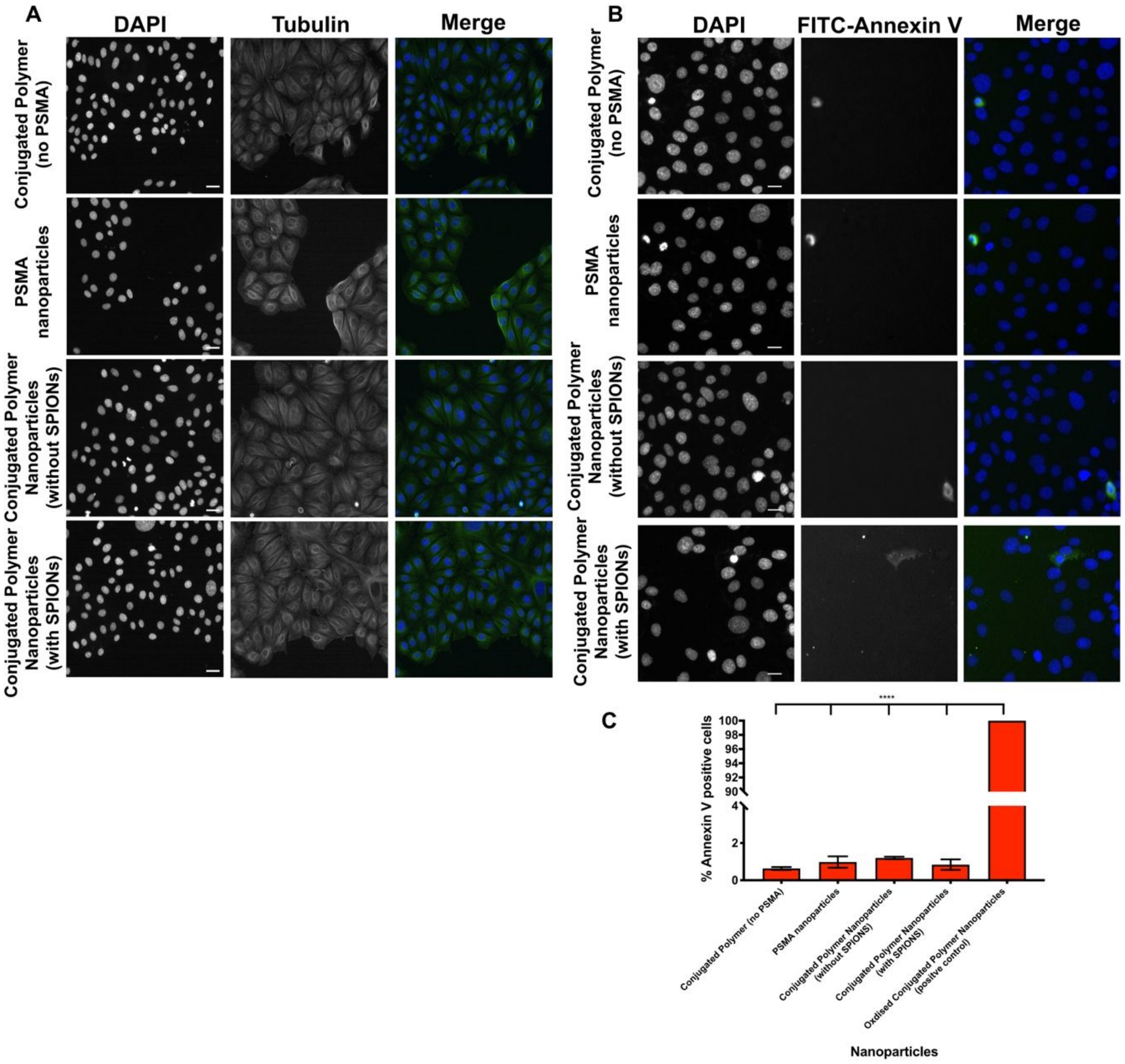

Figure S10. HCE cells treated with conjugated polymer nanoparticles are not showing mitotic arrest or Annexin V positive cells. (A) Representative epifluorescence images of HCE cells fixed and stained with anti-α-tubulin (green) and DAPI (blue) to visualise tubulin and DNA respectively, 24 h after nanoparticle treatment. Conjugated polymer nanoparticles (no PSMA) and PMSA nanoparticles are negative controls. Scale bar = 50 μm. (B) Representative epifluorescence images of HCE cells fixed and stained with Annexin V-FITC (green) and DAPI (blues) to visualise Phosphatidylserine (PS) and DNA, respectively, 24 h after nanoparticles treatment (conjugated polymer nanoparticles (no PSMA) and PMSA nanoparticles. Scale bar = 20μm. (C) Quantification of Annexin V positive cells. Data are presented as mean +/- SEM (n=3), >350 cells were scored per experiment. (**p<0.001).**

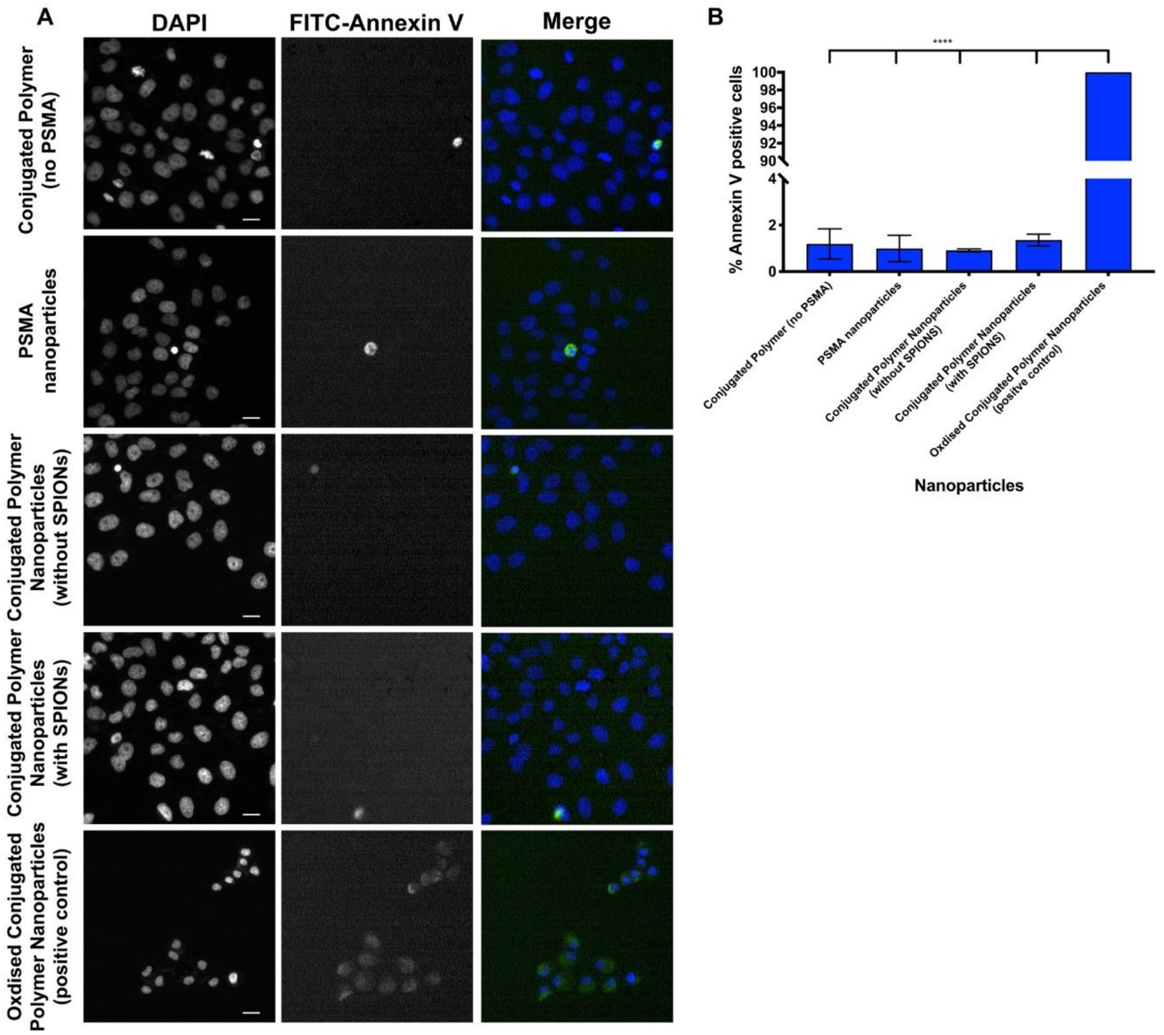

Figure S11. Annexin V is not activated after nanoparticle treatment (A) Representative epifluorescence images of HeLa cells fixed and stained with Annexin V-FITC (green) and DAPI (blue) to visualise phosphatidylserine (PS) and DNA, respectively, 24 h after nanoparticle treatment (Conjugated polymer nanoparticles (no PSMA) and PMSA nanoparticles are negative controls and oxidised conjugated polymer nanoparticles is a positive control, as prepared from [1]). Scale bar = 20 µm. (B) Quantification of Annexin V positive cells. Data are presented as mean +/- SEM (n=3), >350 cells were scored per experiment. (**p<0.001).**

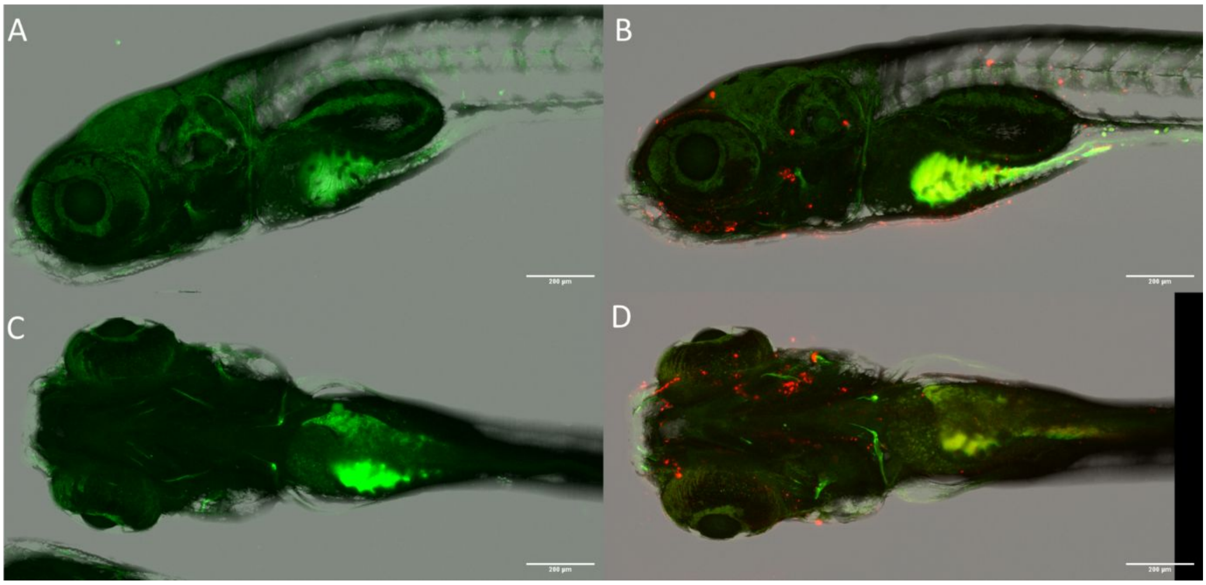

Figure S**12. Images of zebrafish larvae (5 days post fertilisation). A) C) show lateral and ventral sides of larvae untreated with CPNs. B) D) show lateral and ventral sides of larvae treated with CPNs (no SPIONs) at 5 ug/mL. Scale bar is 200 µm.**

References

(1) Bourke, S.; Teijeiro Gonzalez, Y.; Donà, F.; Panamarova, M.; Suhling, K.; Eggert, U.; Dailey, L. A.; Zammit, P.; Green, M. A. Cellular Imaging Using Emission-Tuneable Conjugated Polymer Nanoparticles. *RSC Advances*. 2019, pp 37971–37976.
